# How well do cognitive and environmental variables predict active commuting?

**DOI:** 10.1186/1479-5868-6-12

**Published:** 2009-03-06

**Authors:** Mélanie Lemieux, Gaston Godin

**Affiliations:** 1 Department of Social and Preventive Medicine, Division of Kinesiology, Laval University, Québec, Canada; 2 Research Group on Behaviour in the Field of Health, Laval University, Québec, Canada; 3 Canada Research Chair on Behaviour and Health, Faculty of Nursing, Pavillon Ferdinand-Vandry, 1050 rue De la Médecine, 3e étage, Laval University, Québec, G1V 0A6, Canada

## Abstract

**Background:**

In recent years, there has been growing interest in theoretical studies integrating cognitions and environmental variables in the prediction of behaviour related to the obesity epidemic. This is the approach adopted in the present study in reference to the theory of planned behaviour. More precisely, the aim of this study was to determine the contribution of cognitive and environmental variables in the prediction of active commuting to get to and from work or school.

**Methods:**

A prospective study was carried out with 130 undergraduate and graduate students (93 females; 37 males). Environmental, cognitive and socio-demographic variables were evaluated at baseline by questionnaire. Two weeks later, active commuting (walking/bicycling) to get to and from work or school was self-reported by questionnaire. Hierarchical multiple regression analyses were performed to predict intention and behaviour.

**Results:**

The model predicting behaviour based on cognitive variables explained more variance than the model based on environmental variables (37.4% versus 26.8%; Z = 3.86, *p *< 0.001). Combining cognitive and environmental variables with socio-demographic variables to predict behaviour yielded a final model explaining 41.1% (*p *< 0.001) of the variance. The significant determinants were intention, habit and age. Concerning intention, the same procedure yielded a final model explaining 78.2% (*p *< 0.001) of the variance, with perceived behavioural control, attitude and habit being the significant determinants.

**Conclusion:**

The results showed that cognitive variables play a more important role than environmental variables in predicting and explaining active commuting. When environmental variables were significant, they were mediated by cognitive variables. Therefore, individual cognitions should remain one of the main focuses of interventions promoting active commuting among undergraduate and graduate students.

## Background

Obesity has reached endemic proportions, affecting developed and developing countries [1]. Unfortunately, obese individuals are more likely to develop co-morbidities, such as type 2 diabetes, hypertension and cardiovascular diseases [2,3]. Thus, it is urgent to take action and improve public health. Given that physical activity is known to contribute to maintaining a healthy weight [4], it is important to promote a physically active lifestyle. This could be accomplished by integrating active commuting into people's daily routine. However, in the North American population, only a small proportion of individuals uses walking and bicycling as a mode of transport [5]. Therefore, the purpose of this study was to gain information on the determinants of active commuting in order to guide promotional campaigns. This was accomplished in reference of the theory of planned behaviour (TPB) since this theory has proven efficient in predicting health-related behaviours [6,7]. The advantage of this theory is also that it allows testing of the contribution made by other variables, such as environmental variables, in the prediction of behaviour either as direct determinants or as moderators of the intention-behaviour relationship [8].

### Theory of Planned Behaviour

The TPB suggests that behaviour is predicted by intention and perceived behavioural control (PBC) [8,9]. Intention represents the level at which an individual is ready to act, whereas PBC corresponds to the individual's perceived ability to accomplish the behaviour. The TPB also suggests that intention is predicted by PBC, attitude and subjective norm. Attitude corresponds to the positive or negative value that a person associates with the performance of a given behaviour, whereas subjective norm represents the social pressure felt whether or not to engage in the behaviour. Thus, someone with a favourable attitude who perceives approval from significant others and has a high PBC is more likely to have high intentions to perform the behaviour. The TPB is also open to consideration of other variables if there is evidence that they could be important dimensions. In this regard, there is growing evidence that environmental factors (perceived or real) are associated with the regular practice of physical activity [10-12]. The built environment refers to the presence of physical activity equipment, facilities or infrastructures in one's environment. The built environment may also interact with intention in the prediction of behaviour [13,14].

### Determinants of Active Commuting

To date, only a small number of studies have investigated the cognitive determinants of active commuting, and variations in findings have been reported. Some authors have reported significant associations between walking/bicycling for transport/leisure and intention [15] and PBC [16], whereas others have not observed these associations [15,17].

Likewise, growing interest has been paid to environmental variables and it has been suggested that these variables should be considered in the promotion of physical activity [18]. One of the most promising environmental concepts in active commuting is walkability [19-21]. Walkable neighbourhoods are characterized by high residential density, high land use mix and good connectivity [21]. Others have also reported walking and bicycling facilities such as sidewalks and bicycle paths to be related to active transport [16,22,23]. In contrast, car accessibility was associated negatively with active transport [22,24-26]; people who did not have access to a car were more inclined to use an active transport mode.

Recently, Sallis, Cervero, Ascher, Henderson, Kraft and Kerr [27] suggested a more holistic approach and expressed the opinion that the promotion of physical activity should be based on ecological models that consider the individual, the social environment, the physical environment and policies. As such, it is appropriate to consider individual and environmental variables in the same model when predicting active commuting. However, to date, only a few studies have adopted this approach in the study of physical activity [13,14,28,29] including active transport [30]. In this latter study, walking for transport was correlated with social (family social support) and environmental variables (street connectivity and coastal neighbourhood), whereas cognitions were not significant correlates of active transport. However, these observations were based on cross-sectional data. Other studies have taken intention as the dependant variable. For instance, Eves, Hoppea and McLaren [15] studied walking and bicycling for leisure and transport and determined that walking intentions were predicted by PBC only, whereas bicycling intentions were predicted by attitude and PBC. Similarly, Rhodes and collaborators investigated the role of cognitive and environmental variables on leisure-time walking intentions [13,14]. In their studies, walking intentions were predicted by attitude and social norm. None of the environmental variables contributed to the prediction. Obviously, more studies are needed before a definite conclusion can be reached on the determinants of active commuting.

Thus, the aims of this study were: 1) to identify cognitive and environmental variables predicting active commuting; 2) to identify cognitive and environmental variables explaining intentions to adopt active commuting; 3) to test if environmental variables are mediated by cognitive variables in the prediction of behaviour and intentions; and 4) to test if environmental variables moderate the intention-behaviour relation.

## Methods

### Population and Sample

Participants were students at one of the major universities in the Province of Quebec during the fall term of 2007. We first contacted a few professors known to the authors who were teaching during the term. For those who agreed, all of their students attending the lecture at the time of the baseline survey were asked to participate. Students who agreed to do so were included in the study. A total of 256 undergraduate and graduate students from different academic programs (130 in Nutrition; 36 in Administration; 90 in Nursing) were contacted, and 161 agreed to participate (response rate of 62.9%). Of these 161 respondents, 134 successfully completed the follow-up measure two weeks later (response rate of 83.2%). Four respondents were excluded because it was not possible to match pre- and post-questionnaires. Thus, data from 130 participants were retained for the analysis.

### Procedure for Data Collection

A prospective design was adopted for this study. Students were contacted in class and invited to participate in the study. The study was first explained, and those who agreed to participate had to complete a baseline questionnaire assessing environmental, cognitive and socio-demographic variables. At two-week follow-up, a self-report of use of active commuting frequency (i.e. walking and bicycling to get to and from work or school) was obtained. The project received the approval of the ethics committee of the local university.

### The Baseline Questionnaire

The baseline questionnaire contained five sections: 1) neighbourhood environment; 2) physical activity habits; 3) variables from the theory of planned behaviour (TPB) and related cognitions; 4) habit of using active commuting; and 5) socio-demographic variables [see Additional files 1, 2 and 3].

#### Neighbourhood Environment

As suggested by the International Physical Activity & the Environment Network (IPEN), the Neighbourhood Environment Walkability Survey (NEWS) was used to assess environmental variables related to using walking and bicycling for commuting. It is also considered a reliable and valid instrument [31-34]. However, a modified version was used in order to adjust the instrument to the local environmental context. Thus, the environmental aspects assessed were: a) residential density; b) land use mix (diversity); c) walking/bicycling facilities; d) traffic safety; and e) connectivity. Five-point scales were used to standardize measurement levels for all variables studied. A question regarding access to a motorized vehicle was also added to the questionnaire. Finally, participants were asked how long they had been living in their current neighbourhood. This measure was taken to verify if participants had a good knowledge of their neighbourhood.

#### Theory of Planned Behaviour

The guidelines provided by Ajzen [9] were followed to select items to assess the main variables of the TPB. Each variable was assessed using three to five items (five-point scales) and the average of the items for each construct was taken as the score in the analysis.

#### Past Behaviour and Habit

Active commuting was defined as an activity performed for at least 10 minutes, as recommended by the Public Health Agency of Canada (PHAC) and the American College of Sports Medicine (ACSM) [35,36]. Thus, frequency of active commuting during the past four weeks was obtained. In addition, the habit of using active commuting was measured in reference to the index of Verplanken and Orbell [37]. This scale includes twelve questions related to frequency, automaticity and self-identity. Verplanken and Melkevik [38] have demonstrated the efficacy of their scale in measuring exercising habit. For the purpose of this study, it was adapted to active commuting.

#### Socio-demographic Factors

Socio-demographic variables assessed included gender, age, working/study status, height and body weight. Height and body weight were used to compute body mass index (BMI).

#### Follow-up Behaviour

At two-week follow-up, participants were asked to report the number of times they had used active commuting to get to and from work or school. The same question used to assess past behaviour at baseline was also used to assess behaviour at follow-up, but in reference to the last two weeks: "*In the past 2 weeks, how many times (for a period of a least 10 minutes) did you walk/bicycle to get to your workplace or your school?"*

### Psychometric Qualities of the Questionnaire

To ensure the psychometric qualities of the study questionnaire, 32 participants not involved in the present study completed a two-week test-retest reliability study. The evaluation of the internal consistency and temporal stability of the variables showed appropriate values [see Additional files 1, 2 and 3].

### Analysis

Pearson correlation coefficients were computed to investigate relations between active commuting (i.e. the number of times walking and bicycling were used to commute during the last 2 weeks), intention and other variables. Items with a correlation of p ≤ .20 were retained and tested in subsequent regression analyses. Two separate regression analyses were first performed to evaluate the efficacy of environmental and cognitive variables, respectively, to predict active commuting. Then, hierarchical multiple regression analyses were performed to predict active commuting. The variables were entered according to the following steps: 1) past behaviour; 2) cognitive variables; 3) environmental variables; 4) socio-demographic variables and 5) habit. After each step, non-significant variables (p > .05) were withdrawn one by one, starting with the less significant, until all variables left in the model were significant. For all regression analyses, past behaviour was first entered in order to control for its effect. Habit was entered in the last step to ascertain its additional contribution to the model. Since the habit index used in this study is not a measure of behavioural frequency, past behaviour and habit can be used independently [39]. Finally, the *Z *test procedure described by Tabachnick & Fidell [40] was employed to determine if there was a significant difference between the variance explained by cognitive and environmental variables, respectively, for the prediction of active commuting. The same approach was applied to predict intention to use active commuting.

The role of intention as mediator in the relationship between environmental variables and active commuting was tested using the method suggested by Baron and Kenny [41]. As intention significantly affects active commuting, environmental variables that significantly affected intention (i.e. the mediator) and active commuting (i.e. the dependant variable) were kept for analysis [42]. We used the SAS bootstrap procedure proposed by Preacher and Hayes [43], where age and habit were considered as covariates. The same strategy was used to determine if environmental variables were mediated through intention by the determinants of intention. Habit was considered as a covariate.

A three-step hierarchical regression was applied, as suggested by Aiken and West [44], to test for the moderating effect of environmental variables on the intention-behaviour relationship. To this end, the variables were first mean-centered [41,44]. A significance level of α = 0.05 was used to define statistical significance.

## Results

### Sample

A total of 130 respondents completed both baseline and follow-up questionnaires. The mean age of the sample was 24.0 ± 4.9 years (range: 19 to 48 years), with a predominance of female participants (71.5%). The majority of the participants studied full time (86.2%) and were also working (60.8%). More than half of the participants (52.3%) always had access to a car. In general, participants self-reported having a healthy weight with an average BMI of 22.0 (SD = 3.4). The majority of participants could get to school (74.6%) or work (73.4%) within a 10-minute walk. Also, the majority had been living in their neighbourhood for more than a year (63.8%). No significant differences were observed between participants who completed and those who did not complete the follow-up questionnaire.

Concerning active commuting at follow-up, participants reported using an active commuting mode an average 6.3 times per week (SD = 5.81). Walking was a more popular activity than bicycling (33.1% no walking; 89.2% no bicycling). However, 36.2% of the respondents used active commuting ten or more times per week to get to and from work or school.

Students had a slightly positive intention to use active commuting in the next two weeks (mean: 3.42 ± 1.45, on a five-point scale). Intention was significantly associated with active commuting (*r *= 0.60, *p *< .001). For environmental variables, active commuting and intention were positively correlated with « apartments » and negatively associated with « detached single-family residence », « time to access services », « time to get to school/work » and « car accessibility ». Concerning socio-demographic variables, only age was negatively associated with active commuting, whereas work status was negatively related to intention.

### Predicting Active Commuting

Regression of active commuting at follow-up on past behaviour and cognitions at baseline showed that 37.4% of the variance in active commuting (*p *< 0.001) was explained by past behaviour (β = 0.19, *p *< 0.05; all standardized betas) and intention (β = 0.51, *p *< 0.001). When habit was added to the model, only intention (β = 0.33, *p *< 0.01) and habit (β = 0.34, *p *< 0.01) remained significant predictors, explaining 38.7% of variance (*p *< 0.001). None of the other cognitive variables reached significance (*p *> 0.05).

Regression of follow-up behaviour on past behaviour and environmental variables at baseline yielded to a significant model explaining 26.8% of variance (*p *< 0.001). Three variables were significant predictors: past behaviour (β = 0.29, *p *< 0.001), time to get to work or school (β = -0.19, *p *< 0.05) and car accessibility (β = -0.26, *p *< 0.01). The latter two environmental variables varied as expected; that is, longer time to get to work or school and having access to a car played a negative role.

The model predicting behaviour based on cognitions was compared with the model predicting behaviour based on environmental variables. The *Z *test showed that the total variance explained by cognitive variables (i.e. past behaviour and intention) was significantly higher than the model of environmental variables (i.e. past behaviour, time to get to school/work and car accessibility) in the prediction of active commuting (Z = 3.86, *p *< 0.001, *N *= 130).

When both cognitive and environmental variables were combined in the same hierarchical linear regression analysis, none of the environmental variables reached significance (*p *> 0.05) (see Table 1). The final model explained 41.1% of the variance in behaviour (active commuting) (*p *< 0.001); the significant determinants were intention (*p *< 0.01), age (*p *< 0.05) and habit (*p *< 0.01).

**Table 1:** Hierarchical multiple regression analysis of active commuting combining cognitive and environmental variables

Variable Entered	Step 1	Step 2	Step 3	Step 4	Step 5	Final
Past behaviour	0.46***	0.19*	0.17*	0.17*	0.14	
Intention		0.51***	0.44***	.50***	0.28*	0.31**
Time to get to school/work			-0.07			
Car accessibility			-0.11			
Study status				-0.06		
Age				-0.16*	-0.16*	-0.17*
Habit					0.30*	0.33**

R^2 ^adjusted	0.21	0.37	0.38	0.40	0.42	0.41
Model F	43.11***	39.51***	20.52***	22.08***	24.58***	30.97***
ΔR^2^	-	0.16***	0.17***	0.19***	0.21***	-

*: p < .05, **: p < .01, ***: p < .001; all standardized betas

### Predicting Intention

Given that intention is the most important predictor of behaviour according to the TPB, analyzing its determinants was justified. When only TPB variables were considered, 72.6% of variance (*F*(3, 126) = 114.85, *p *< 0.001) in intention was explained by PBC (β = 0.60, *p *< 0.001), attitude (β = 0.29, *p *< 0.001) and subjective norm (β = 0.02, *p *= 0.77). When past behaviour was included in the model, intention was predicted by past behaviour (β = 0.14, *p *< 0.01), PBC (β = 0.57, *p *< 0.001) and attitude (β = 0.28, *p *< 0.001); the explained variance was 0.74 (*p *< 0.001). The final model for the cognitive variables explained 78.2% of the variance of intention (*p *< 0.001), with significant determinants being PBC (β = 0.43, *p *< 0.001), attitude (β = 0.17, *p *< 0.05) and habit (β = 0.37, *p *< 0.001).

In the context of environmental variables and controlling for past behaviour (β = 0.27, *p *< 0.001), two factors contributed to the prediction of intention: time to get to work or school (β = -0.27, *p *< 0.001); and car accessibility (β = -0.36, *p *< 0.001). The contribution of these two environmental variables varied as expected, i.e. negatively. This model of the environmental variables explained 40.4% of the variance (*p *< 0.001).

The models predicting intention from cognitions and environmental variables were compared. The *Z *test score (*Z *= 9.51, *p *< 0.001, *N *= 130) showed that the proportion of variance in intention explained by cognitive variables (i.e. past behaviour, PBC and attitude) was significantly greater than the model based on environmental variables (i.e. past behaviour, time to get to school/work and car accessibility).

Combining environmental and cognitive variables indicated that no environmental variable contributed significantly to the prediction of intention (see Table 2). The final model was formed by PBC (*p *< 0.001), attitude (*p *< 0.05) and habit (*p *< 0.001); it explained 78.2% (*p *< 0.001) of the variance in the intention to use active commuting.

**Table 2:** Hierarchical multiple regression analysis of intention combining cognitive and environmental variables

Variable Entered	Step 1	Step 2	Step 3	Step 4	Step 5	Final
Past behaviour	0.44***	0.14**	0.11*	0.13**	0.07	
PBC		0.57***	0.51***	0.50***	0.42***	0.43***
Attitude		0.28***	0.26**	0.27**	0.18**	0.17*
Time to get to school/work			-0.05			
Car accessibility			-0.15**	-0.15**	-0.09	
Work status				-0.07		
Social deprivation				0.04		
Habit					0.30***	0.37***

R^2 ^adjusted	0.19	0.74	0.76	0.75	0.79	0.78
Model F	41.27***	124.63***	82.65***	59.70***	96.93***	154.89***
ΔR^2^	-	0.55***	0.57***	0.56***	0.60***	-

*: p < .05, **: p < .01, ***: p < .001; PBC = perceived behavioural control; all standardized betas

### Mediation Analysis

**Figure 1:**
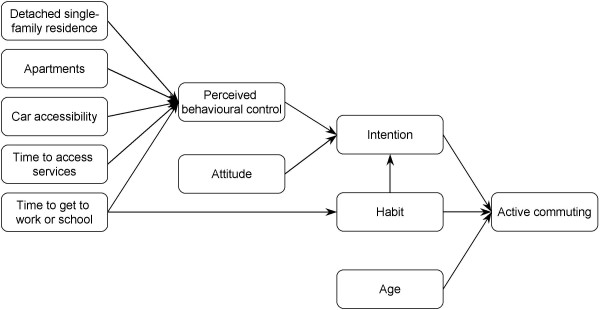
**Observed model for the prediction of active commuting**. Only significant associations are illustrated.

Mediation tests were performed to identify how environmental factors contribute to the formation of intention and prediction of behaviour. Five environmental variables met Baron and Kenny's (1986) criteria and were tested for mediation by intention in the prediction of active commuting, controlling for age and habit. None of these factors reached significance: detached single-family residence (CI_95%_: -0.52, 0.17); apartments (CI_95%_: -0.14, 0.41); time to access services (CI_95%_: -0.04, 0.05); time to get to work or school (CI_95%_: -0.06, 0.002); and car accessibility (CI_95%_: -0.93, 0.02). In fact, mediation analyses indicated that environmental variables were mediated by the determinants of intention. Time to get to work or school was mediated by PBC (CI_95%_: -0.03, -0.0008; covariate: habit) and habit (CI_95%_: -0.13, -0.06), whereas detached single-family residence (CI_95%_: -0.65, -0.13), apartments (CI_95%_: 0.12, 0.67), time to access services (CI_95%_: -0.13, -0.06) and car accessibility (CI_95%_: -1.51, -0.80) were mediated by PBC. No other significant relation was found. Figure 1 illustrates the interrelationship between cognitions and environmental variables in explaining intention and predicting behaviour.

### Analysis for Moderating Effects

Detached single-family residences, apartments, time to access services, time to get to work or school and car accessibility were environmental variables tested as potential moderators of the intention-behaviour relationship, since they were significant correlates of active commuting. However, none of these variables interacted significantly with intention in the prediction of active commuting (all *p *> 0.05).

## Discussion

To our knowledge, this is the first prospective study investigating cognitive and environmental variables to determine their relative contribution to the prediction of active commuting by undergraduate and graduate students to get to and from work or school. Intention, habit and age were identified as significant predictors of active commuting. None of the environmental variables contributed to this prediction.

The observation that intention contributes to the prediction of active commuting is in agreement with previous published meta-analyses showing its role in the prediction of health-related behaviours [7,45,46]. Notwithstanding this observation, the TPB was not fully supported because factors other than intention (or PBC) contributed to the prediction of behaviour.

The most important active commuting predictor observed in this study was habit. People who are in the habit of using active commuting do so more often. This observation is congruent with Triandis' theory of interpersonal behaviour [47] that considers habit as a direct predictor of behaviour. Usually, in scientific literature, habit is assessed as a frequency of past behaviour. However, in the present study, habit was measured using the scale of Verplanken and Orbell [37] as well as the mere measure of frequency of past behaviour. However, when habit and frequency of past behaviour were tested in the same analysis, frequency of past behaviour did not reach significance. This suggests that frequency of past behaviour is mediated by habit. This observation provides additional support to Verplanken and Orbell [37], who consider habit as a cognitive construct. Thus, as proposed by Verplanken and Melkevik [38], the development of habituation should be considered as an intervention goal for active commuting among undergraduate and graduate students. It was reported in a recent Canadian survey [48] that distance separating an individual's home from the workplace or school can be a barrier to active commuting. In this respect, it may be noted that living close to one's workplace/school most likely contributes to the development of the habit of using active commuting, since in the present study the effect of "time to get to work or school" on behaviour was mediated by habit, along with PBC.

Another predictor of active commuting was age. Younger individuals were more likely to use active commuting. Similar findings were reported by Butler, Orpana, and Wiens [49] who suggested that the effect of age was related to "safety" concerns among older individuals. However, this was not confirmed in the present study, since traffic safety did not contribute to the prediction. Given the cultural context of the present study (undergraduate and graduate student population), we are more inclined to believe that older adults are less open to the idea of taking more time to get to work/school. This could be particularly true for older individuals who have to cope with school, work and family duties. Others studies conducted in different cultural and social milieus are needed before a more definite conclusion can be reached.

Finally, cognitive variables were stronger predictors of behaviour than were environmental factors. In fact, none of the environmental variables contributed to the prediction when cognitions were included in the model. This concurs with the work of Giles-Corti and Donovan [29] who found that "individual and social environment determinants outweigh the role played by physical environmental determinants of exercising as recommend" (p.1804). Moreover, none of the environmental variables had moderating effects on the intention-behaviour relationship. This result differs from those previously reported by Rhodes et al. [13,14], who found that "infrastructures proximity" moderate the intention-leisure time walking relation. These differences in findings may be attributed to the type of environmental variables measured, their variation in operationalization and differences in the population under study. It is also conceivable that environmental moderators varied for different types of physical activity. However, these results reveal that cognitions nevertheless have an important place in active commuting promotion, notwithstanding the growing attention given to environmental variables. From an ecological perspective, it is clear that individuals evolve in an environment with which they interact and from which they could be influenced. Nonetheless, it seems that environmental variables can facilitate the behaviour but are insufficient to generate the action [29].

Given that intention was a significant determinant of behaviour among undergraduate and graduate students, it can be used to guide the development of interventions. In this regard, perceived behavioural control, attitude and habit deserve special attention. Perceived behavioural control was found to be the most important determinant of intention. This means that students who had a high perception of behavioural control also had a high intention to use active commuting. Similar results have been observed for physical activity [14,15,46,50]. In the context of the present study, it may also be proposed that PBC is partly defined by residential density (detached single-family residence; apartments), land use mix (time to access services; time to get to work/school); and car accessibility, since their effect on intention was mediated by PBC.

Attitude was another factor positively associated with the intention to use active commuting among undergraduate and graduate students. Thus, someone with a positive outlook towards active commuting is more inclined to use it. The meta-analysis of Downs and Hausenblas [50] on exercising supports this view. In their review, they noted that attitudes were as important as PBC in the prediction of intention. However, in the present study, the role of attitude was less important than the contribution of PBC, which is in line with the meta-analysis of Hagger, Chatzisarantis, and Biddle [46]. These variations in relative importance suggest that the contribution of the determinants can vary according to the type of physical activity under study (e.g. active commuting versus leisure-time physical activity).

Regarding the contribution of habit, this point was discussed previously with respect to behaviour prediction. The only additional point that needs to be mentioned is the fact that several authors have previously reported that habit contributes to the prediction of intention [15,46].

### Limitations and Strengths

Some limitations in the present study should be mentioned. First, the study population consisted of undergraduate and graduate students. More females (71.5%) than males participated. Therefore, the present observations cannot be extended to the general population. In addition, the sample size was modest (N = 130). Notwithstanding this point, we obtained a power of 100% for our final model predicting behaviour. Participants were volunteers, consequently more likely to be interested in the studied behaviour. Also, an objective measure of active commuting would have been preferable to a self-report measure, although our measure presented good test-retest value. The inclusion of objective measures of environmental variables would also have been interesting, since it was recently documented that objective and subjective environmental measurements are not necessarily equivalent [51]. Notwithstanding this latter observation, the environmental questions used in this study have been validated in previous studies [31,32,34]. Nonetheless, additional work is needed on this topic, since certain difficulties were encountered translating a number of items into French. To be more effective, more reliable tools standardizing the definitions of environmental variables should be developed.

Compared to previously published work, one of the major strengths of this study is its use of a prospective design, allowing behaviour prediction. Moreover, as it has been suggested in the past [52], our study investigated a specific type of physical activity, given that different environmental variables seem to be associated with transportation and recreational physical activity. Another salient point in this research was the consideration of cognitive and environmental variables simultaneously. A further innovative element was to define active commuting habit as a cognitive variable evaluated with the validated Self-Report Habit Index [37,38] instead of measuring frequency of past behaviour alone.

## Conclusion

In summary, intention, habit and age predicted behaviour significantly. These results indicate, along with the findings of Giles-Corti and Donavan [29], that cognitive variables play a more important role in the prediction of active commuting than do environmental variables. This reinforces the opinion of Giskes, Kamphuis, van Lenthe, Kremers, Droomers and Brug [53] that it is premature to encourage extensive investment in environmental interventions and that more studies are needed to determine a standardized and validated method to operationalize environmental variables. Until then, interventions promoting active commuting among undergraduate and graduated students should focus on developing a high intention to use active commuting based on the development of perceived behavioural control and attitude. In addition, some consideration should be given to residential density, land use mix and car accessibility, as their influence is mediated by the cognitions explaining intention to use active commuting.

## Competing interests

The authors declare that they have no competing interests.

## Authors' contributions

ML carried out the study, performed the statistical analysis and drafted the manuscript. ML and GG participated in the study design. GG revised critically the manuscript. All authors read and approved the final manuscript.

## Supplementary Material

Additional file 1 **Environmental variables: Questions, scales and psychometric qualities of the survey**. Table presenting all the questions and answer scales used in the baseline survey and the psychometric values obtained at the test-retest study. Items used to assess the environmental variables and their psychometric qualities. Click here for file

Additional file 2 **Behaviour, habit and cognitive variables: Questions, scales and psychometric qualities of the survey.** Table presenting all the questions and answer scales used in the baseline survey and the psychometric values obtained at the test-retest study. Items used to assess behaviour, habit and cognitive variables and their psychometric qualities. Click here for file

Additional file 3 **Behaviour, habit and cognitive variables: Questions, scales and psychometric qualities of the survey (continued). **Table presenting all the questions and answer scales used in the baseline survey and the psychometric values obtained at the test-retest study. Items used to assess behaviour, habit and cognitive variables and their psychometric qualities. Click here for file
