# Supplementary material for: How well do cognitive and environmental variables predict active commuting?
Source: Int J Behav Nutr Phys Act. 2009 Mar 6;6:12. doi: 10.1186/1479-5868-6-12 (PMC2667470; doi:10.1186/1479-5868-6-12)
Supplement: Additional file 1 — Environmental variables: Questions, scales and psychometric qualities of the survey. Table presenting all the questions and answer scales used in the baseline survey and the psychometric values obtained at the test-retest study. Items used to assess the environmental variables and their psychometric qualities. [file 1479-5868-6-12-S1.pdf]

Appendix 1

Environmental Variables: Questions, Scales and Psychometric Qualities of the Survey

Item	Scale	α_2	r
**Variable**			
**Availability of sidewalks**			
▪ There are sidewalks on most of the streets in my neighbourhood.	Strongly disagree/ strongly agree	---	0.49[1]
**Availability of bicycle lanes**			
▪ There are bicycle lanes on most of the streets in my neighbourhood.	Strongly disagree/ strongly agree	---	0.68[1]
▪ It is safe to ride a bicycle in or near my neighbourhood.			0.69[1]
**Perceived safety from traffic**			
▪ There is so much traffic along the street I live on or nearby that it makes it difficult or unpleasant to walk in my neighbourhood. (Reversed)	Strongly disagree/ strongly agree	---	0.36[1]
▪ The speed of traffic on the street I live on nearby is usually slow (50 km/h or less).			0.57[1]
▪ Most drivers exceed the posted speed limits while driving in my neighbourhood. (Reversed)			0.66[1]
▪ When walking in my neighbourhood, there are a lot of exhaust fumes (such as from cars, buses). (Reversed)			0.57[1]
**Connectivity**			
▪ The street design in my neighbourhood has few, if any, dead ends.	Strongly disagree/ strongly agree	---	0.44[1]
▪ There are many four-way intersections in my neighbourhood.			0.72[1]
**Residential density**			
▪ How common is each type of residence listed below in your immediate neighbourhood?	None/some/a lot	---	
Detached single-family residence			0.88[1]
Row house			0.44[1]
Apartment			0.79[1]
**Diversity**			
▪ About how long would it take to get from your home to the nearest businesses or facilities listed below if you walked to them? grocery store, shopping centre, post office, library, bank/credit union, video store, bus stop, work, school	1-5/6-10/11-20/21-30/>30 minutes	---	0.49-0.85[1]
**Time in the neighbourhood**			
▪ How long have you lived in your current neighbourhood?	≤ 3months /4-12 months/> 1 year	---	0.87[1]

[1] = Kappa
α = Cronbach's alpha coefficient (main study)
r = Intraclass correlation coefficient (test-retest)
