# Supplementary material for: How well do cognitive and environmental variables predict active commuting?
Source: Int J Behav Nutr Phys Act. 2009 Mar 6;6:12. doi: 10.1186/1479-5868-6-12 (PMC2667470; doi:10.1186/1479-5868-6-12)
Supplement: Additional file 2 — Behaviour, habit and cognitive variables: Questions, scales and psychometric qualities of the survey. Table presenting all the questions and answer scales used in the baseline survey and the psychometric values obtained at the test-retest study. Items used to assess behaviour, habit and cognitive variables and their psychometric qualities. [file 1479-5868-6-12-S2.pdf]

Appendix 2

Behaviour, Habit and Cognitive Variables: Questions, Scales and Psychometric Qualities of the Survey

Item	Scale	α_2	r
**Physical Activity**			
**For commuting**			
▪ In the past 4 weeks, how many times (for a period of at least 10 minutes) did you walk to get to your workplace or your school?	One way<br>Return	---<br>---	0.78<br>0.78
▪ In the past 4 weeks, how many times (for a period of at least 10 minutes) did you bicycle to get to your workplace or your school?	One way<br>Return	---<br>---	0.98<br>0.98
**Theory of Planned Behaviour (during the next two weeks)**			
**Attitude**			
▪ For me, to use regularly active commuting would be…	Very unsatisfying/ very satisfying<br>Very tiring/ very energizing<br>Very unpleasant/ very pleasant<br>Very disadvantageous/ very advantageous<br>Very useless/ very useful	0.91	0.72
**Subjective norm**			
▪ If I were to use active commuting regularly, most of the people who are important to me would…<br>▪ Most of the people who are important to me would recommend that I use active commuting on a regular basis.<br>▪ The people who are most important to me think I should use active commuting on a regular basis.	Strongly disapprove/ strongly approve<br>Strongly disagree/ strongly agree	0.87	0.83
**Perceived behavioural control**			
▪ I think I am able to use active commuting on a regular basis.<br>▪ It is up to me to use active commuting on a regular basis.<br>▪ For me, regular use of active commuting would be…<br>▪ If I wanted, I could use active commuting on a regular basis.	Strongly disagree/ strongly agree<br>Very difficult/ very easy<br>Very unlikely/ very likely	0.90	0.94
**Intention**			
▪ I intend to use active commuting on a regular basis.<br>▪ I will try to use active commuting on a regular basis.<br>▪ I will use active commuting on a regular basis.	Very unlikely/ very likely	0.98	0.84

[1] = Kappa
α = Cronbach's alpha coefficient (main study)
r = Intraclass correlation coefficient (test-retest)
