# Supplementary material for: How well do cognitive and environmental variables predict active commuting?
Source: Int J Behav Nutr Phys Act. 2009 Mar 6;6:12. doi: 10.1186/1479-5868-6-12 (PMC2667470; doi:10.1186/1479-5868-6-12)
Supplement: Additional file 3 — Behaviour, habit and cognitive variables: Questions, scales and psychometric qualities of the survey (continued). Table presenting all the questions and answer scales used in the baseline survey and the psychometric values obtained at the test-retest study. Items used to assess behaviour, habit and cognitive variables and their psychometric qualities. [file 1479-5868-6-12-S3.pdf]

Appendix 2

Behaviour, Habit and Cognitive Variables: Questions, Scales and Psychometric Qualities of the Survey (continued)

Item	Scale	α_2	r
**Habits**			
▪ Using active commuting is something:<br>– I do frequently.<br>– I do automatically.<br>– I do without having to consciously remember.<br>– that makes me feel weird if I do not do it.<br>– that would require effort not to do it.<br>– that belongs to my daily routine.<br>– I start doing before I realize I'm doing it.<br>– I would find hard not to do.<br>– I have no need to think about doing.<br>– that is typically "me".<br>– I have been doing for a long time.	Strongly disagree/ strongly agree	0.96	0.96

[1] = Kappa
α = Cronbach's alpha coefficient (main study)
r = Intraclass correlation coefficient (test-retest)
